# Partial Infarction of Cecal Wall Presenting as Acute Appendicitis

**DOI:** 10.7759/cureus.31408

**Published:** 2022-11-12

**Authors:** Vugar Suleimanov, Farrukh Shahzad

**Affiliations:** 1 Surgery, Jubail General Hospital, Jubail, SAU; 2 General Surgery, Al Razi Hospital, Lahore, PAK

**Keywords:** splanchnic vasoconstriction, low flow state, ishcemic colitis, acute appendicitis, isolated cecal necrosis

## Abstract

Acute appendicitis is one of the most common causes of acute abdomen in children and young adults, with differential diagnoses involving several surgical, medical as well as obstetrics and gynecology (OB-GYN)-related conditions. Cecal wall necrosis is not usually included in the differential diagnosis of acute appendicitis, mainly because of the rare nature of the condition and the relatively low level of awareness among surgeons. We present a case of isolated cecal wall necrosis presenting as acute appendicitis. It involves a 42-year-old male patient who presented to the emergency department (ED) of our hospital with abdominal pain for three days. The pain was felt over the right lower quadrant and was associated with nausea, vomiting, and anorexia. The case was diagnosed as acute appendicitis and the patient was taken to the operating room (OR). Upon entering the abdomen, necrosis of the anterolateral wall of the cecum was discovered with a normal appendix. Resection of the cecum was performed, followed by ileocolic anastomosis. The patient was discharged in good condition after 10 days of hospital stay.

This case report highlights the importance of considering cecal wall necrosis in patients presenting with right lower quadrant abdominal pain who display atypical signs and symptoms of appendicitis; we also wish to promote awareness among surgeons about this rare condition.

## Introduction

Right-sided ischemic colitis is considerably less common than left-sided ones. Isolated necrosis of the cecum is quite rare, comprising only 2.2% of the total cases of ischemic colitis [1]. A few cases of isolated cecal necrosis (ICN) have been reported in the literature, most of them involving elderly patients with multiple comorbidities [2], causing "low flow state" such as heart failure, cardiac surgery, shock, hemodialysis, etc, and a few due to GI malignancy [3,4]. Isolated cecal wall ischemia/necrosis can occur due to both occlusive and non-occlusive events, as hypothesized in the article by Weisner et al. [5]. It seems that anatomic variations of cecal blood supply may increase the propensity of some individuals to develop isolated cecal ischemia, where anterior and posterior cecal arteries arise from the colic branch of the ileocecal artery, rather than from the arcade between the colic and ileal branches of the ileocecal artery [5,6]. Preoperative diagnosis is usually difficult to make unless contrast-enhanced CT (CECT) is used. However, most cases of acute appendicitis do not require CECT, unless the diagnosis is in doubt. In this report, we discuss a case of ICN, which was preoperatively diagnosed as a case of acute appendicitis.

## Case presentation

**Figure 1:**
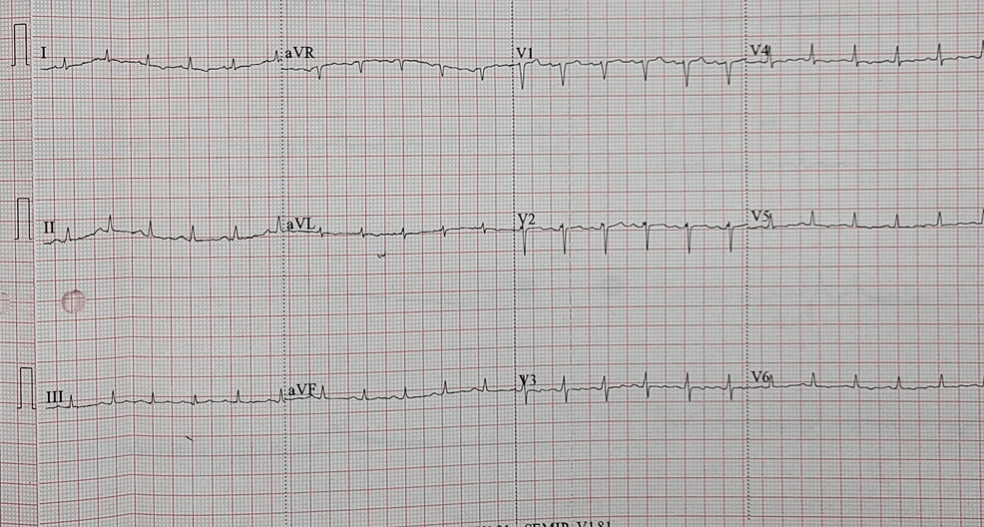
Low voltage ECG showing sinus tachycardia ECG: electrocardiogram

A 42-year-old male patient presented to the emergency department (ED) of our hospital with abdominal pain lasting three days, which was associated with nausea, vomiting, anorexia, diaphoresis, and bluish discoloration of the tips of his fingers and toes. The pain, which was persistent and of moderate intensity, was localized to the right lower quadrant since the onset. The patient denied any significant illness in the past or any use of prescription or illicit drugs. He had experienced no similar complaints before. On examination, it was noted that the patient had a significantly elevated heart rate of 144/min, blood pressure of 106/66 mmHg, a core body temperature of 37.1 ℃, respiratory rate of 20/min, and SPO_2_ of 97% on room air. His heart sounds were muffled on auscultation of the chest, and the abdomen was very tender over the right lower quadrant with positive rebound tenderness. Laboratory investigations (Table 1) showed leukocytosis with left shift, mild renal impairment, lactic acidosis, and elevated creatine kinase (CK) and cardiac enzymes. Low voltage ECG (Figure 1) showed sinus tachycardia, but no signs of ischemia.

**Table 1:** Laboratory investigations

Name of the test	Result	Reference range
Leukocytes	16,000	4,000–10,500
Neutrophils	89%	40–62%
Creatinine	128	53–115 umol/L
Blood urea nitrogen (BUN)	15.1	2.5–6.9 mmol/L
Potassium	5.25	3.5–5.1 mmol/L
Lactic acid	7.75	0.4–2 mmol/L
Creatine kinase (CK)	1,135	26–308 U/L
Creatine kinase MB (CKMB)	104.6	7–25 ng/ml
Troponin T	0.718	0–0.06 ng/ml
Serum PH	7.27	7.35–7.45

**Figure 2:**
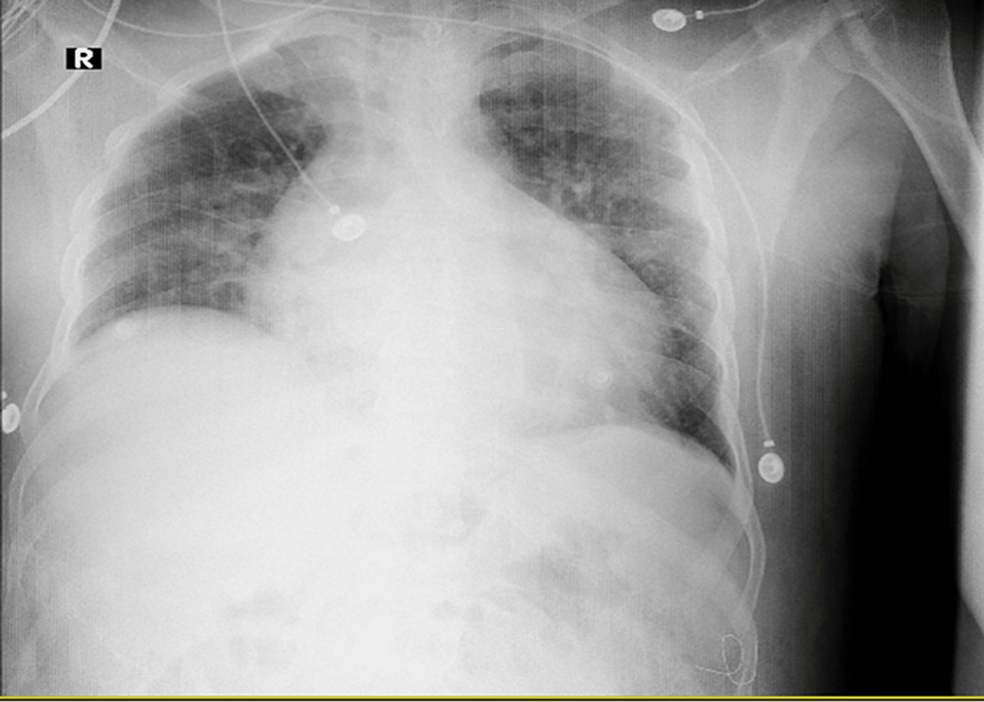
Chest X-ray showing enlarged cardiac silhouette

A chest X-ray (Figure 2) showed an enlargement of the cardiac silhouette, which proved to be due to cardiomegaly by a cardiac echo. Echocardiography showed pericardial effusion without signs of tamponade and no wall motion abnormalities.

**Figure 3:**
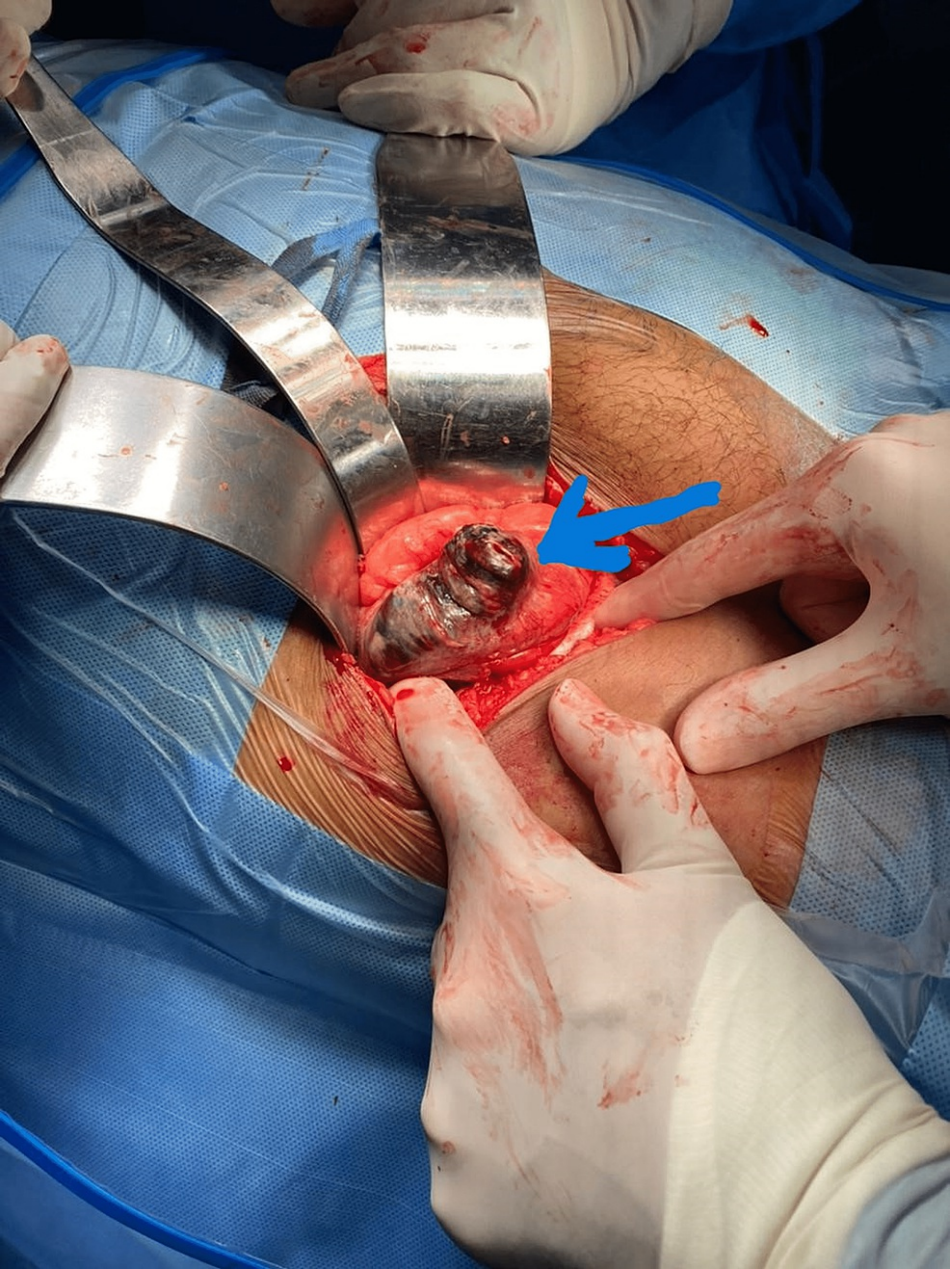
Intraoperative image showing necrosis of the anterolateral cecal wall (blue arrow)

The abdominal ultrasound exam was unremarkable, except for bilateral mild pleural effusion. The appendix could not be visualized due to a large amount of bowel gas present in the right lower quadrant. A CECT was requested, but unfortunately could not be performed, because the only scanner in the hospital was out of order on that day. The Alvarado Score (Table 2) was 8 out of 10, making the diagnosis of acute appendicitis probable. It was assumed that the patient had sepsis due to acute appendicitis (although there were multiple findings suggesting that some other pathology could also be at play). A sepsis resuscitation bundle was initiated; the patient was resuscitated with intravenous (IV) Ringer’s lactate solution (30 ml/kg), a blood sample was taken for a culture/sensitivity test, and broad-spectrum antibiotics were started. The patient was then taken to the operating room (OR) for source control. Upon entering the abdomen through a grid-iron incision, we found that the appendix was normal. However, there was a patch of necrosis over the anterolateral cecal wall measuring about 10 x 5 cm (Figure 3).

The incision was extended, the cecum was resected, and a side-to-side anastomosis between the terminal ileum and ascending colon was created by a linear 3.5-mm stapler. Limited exploration did not reveal any other abnormality. Postoperatively, the patient was managed in the ICU. His condition kept improving day by day. Acidosis resolved, renal and liver function tests normalized by day three postoperatively, and so did his vital signs. Repeat echocardiography showed minimal pericardial effusion only. His peripheral cyanosis improved except on the right upper limb. Right upper limb arterial and venous Doppler studies did not reveal any abnormality. The patient was shifted to the floor on the third day after surgery, and oral feeding was initiated gradually, which was well tolerated.

The patient developed a wound infection on day six, which was treated by routine wound care. Histopathological analysis demonstrated isolated transmural cecal necrosis with marked infiltration of the cecal wall by numerous bacteria and neutrophils. No pathological changes were observed in the margins of the divided cecum and ileum. The appendix too was free of pathology. On the 10th day of admission, the patient was discharged home in satisfactory condition. He was followed up in the outpatient clinic 10 days after the discharge. Repeat cardiac echo showed complete resolution of the pericardial effusion, and the wound was about to close; additional tests to rule out vasculitis and connective tissue diseases were normal except for a positive antinuclear antibody (ANA) test, which was not enough by itself to provide any conclusive impression. All other lab tests were normal (renal, liver functions, complete blood count, electrolytes, etc.). The patient did not have cyanosis on his fingertips anymore, but he did have dry gangrene on the tip of his right little finger. After six months, the patient was seen in the outpatient clinic again; he was found to be all right from all aspects, but the tip of his right little finger had autoamputated.

## Discussion

Ischemic colitis and colonic infarction usually affect the left colon in a segmental fashion due to the somewhat compromised circulation in "watershed areas", including the splenic flexure (Griffith’s point) and rectosigmoid junction (Sudeck’s point) [5]. ICN, on the other hand, is a rare and relatively less well-known entity.

It is widely believed that a "low flow state" in elderly patients with significant comorbidities, such as heart failure, shock, cardiac surgery, and hemodialysis, is the major contributing factor in ischemic colitis affecting the left colon, and the same mechanism is proposed by many authors as a cause of ICN too [5,7,8,9]. Interestingly, we did not find any case reports where a low flow state has caused left-sided ischemic colitis and cecal necrosis in the same patient, which suggests that a low flow state alone is not enough to cause cecal necrosis.

According to Athanasiou et al., the cecum is mainly supplied by the anterior and posterior cecal arteries [8]. These are terminal branches that arise from the ileal or the colic branch of the ileocolic artery. It is hypothesized that the absence of a vascular arcade between the ileal and colic branches in some individuals makes the cecum vulnerable to ischemia. Moreover, it has been suggested that anatomic variations, such as an absence of the posterior or anterior cecal artery, may also play an important role in the pathogenesis of ICN. It is reasonable to believe that ICN affects those individuals with compromised cecal blood supply due to anatomic variations, who are subject to a low flow state, which could be due to shock, heart failure, hemodialysis, etc. Although our patient had a negative past medical history and was relatively young, he did have systemic vasoconstriction with peripheral cyanosis affecting all of his limbs. It is highly likely that his partial cecal infarction was due to splanchnic vasoconstriction and the presence of deficient cecal blood supply, and probably an absent anterior cecal artery (patchy infarction involving the anterolateral aspect of the cecum).

Cecal infarction typically presents with abdominal pain, which starts over the right iliac fossa and remains there, and is frequently associated with leukocytosis, nausea, vomiting, and anorexia, just like in acute appendicitis [6-7,10]. Some patients develop fever too. If a CT is performed before surgery, cecal necrosis is typically seen as isolated, low-attenuating circumferential cecal wall-thickening [5]. There may also be some pericecal inflammation, stranding of mesenteric fat, ascites, or even free intraperitoneal air if a patient presents late or if the diagnosis is delayed. Frequently, such cases become difficult to differentiate from complicated appendicitis, if the findings are observed around the appendix. Although a significant number of cecal necrosis have been diagnosed by CT in reported cases [8-9,11], many cases are diagnosed during surgery due to the difficulty in diagnosis even after performing CECT, especially if cecal perforation has already taken place. We do not recommend using CT for all cases of suspected appendicitis, because it involves radiation exposure, increased cost, and potential contrast-related complications; moreover, it is not widely available, especially in developing and less developed countries.

The Alvarado Score (Table 2), on the other hand, is a useful tool to make a decision related to diagnosis, if the patient presents with symptoms and signs suggestive of acute appendicitis [12].

**Table 2:** The Alvarado Score* *[12]

Signs	Score
Right lower quadrant tenderness	2
Fever (>37.5 °C)	1
Rebound tenderness	1
Symptoms	
Anorexia	1
Nausea or vomiting	1
Migration of pain to the right iliac fossa	1
Laboratory values	
Leukocytosis (>10,000)	2
Neutrophils (>75%)	1

If the Alvarado Score is 3 or 4, acute appendicitis is unlikely. While scores of 5-6 point to likely acute appendicitis, a CT is recommended, whereas scores of 7 or more indicate that the diagnosis of acute appendicitis is probable and surgical consultation is needed in an emergency department setting, according to the study by McKay et al. [13]. Our patient had an Alvarado Score of 8, making the diagnosis of acute appendicitis probable. However, in elderly patients with comorbidities and in younger patients with an atypical presentation, especially when some of the signs and symptoms do not add up, the scoring must be used with caution, and we do recommend using CECT in such cases.

Diagnostic laparoscopy is also considered a useful option to make a definitive diagnosis and choose a surgical strategy that includes the incision type [14]. Most cases of ICN can be managed by laparoscopic resection of the cecum if the surgeon is skilled enough. Early diagnosis and expeditious surgical management can lead to favorable outcomes in most cases of ICN.

## Conclusions

Isolated cecal wall necrosis should be included in the differential diagnosis for right lower quadrant pain, especially in elderly patients with comorbidities that cause a "low flow state" and in younger patients who display signs and symptoms not fully compatible with acute appendicitis. In such circumstances, CECT is a highly recommended diagnostic modality, which can help to establish a diagnosis preoperatively and determine the appropriate access to the site of pathology. If the diagnosis cannot be made even after performing a CT, diagnostic laparoscopy should be considered.
